# ENGAGE UNDERREPRESENTED GROUPS IN TECHNOLOGY AND AGING RESEARCH: LIVED EXPERIENCE AT GEOGRAPHICALLY DIVERSE SITES

**DOI:** 10.1093/geroni/igad104.1457

**Published:** 2023-12-21

**Authors:** Jeffrey Kaye, Zach Beattie

## Abstract

Minoritized and marginalized older adults face multifaceted barriers that could prevent them from participating in aging research, including but not limited to not having access to research opportunities, time and transportation constraints, lack of trust with health care providers and researchers, and language barriers. When the research projects involve technology, whether as a tool for engagement or recruitment, intervention delivery, or outcome monitoring and assessment, additional challenges exist in recruiting underrepresented groups. The technology adoption gap has been widely reported. Minoritized and marginalized older adults are less likely to have internet access or feel less confident in using technology compared to their more privileged counterparts. This symposium introduces the lived experience and lessons learned from engagement and recruitment of underrepresented populations at multiple Alzheimer’s Disease Research Centers. The first presentation presents outreach and recruitment efforts at the Oregon Outreach, Recruitment & Education Core. The authors will discuss strategies and benefits to engage people of underrepresented backgrounds in the research workforce. The following two presentations focus on recruitment efforts for installing an in-home monitoring technology platform for diverse older adult populations, including African Americans in Chicago, IL, and Hispanic/Latino immigrants in San Antonio, TX. The fourth presentation describes the response, retention, and enrollment rate of an online dementia research registry and the effort to adapt the registry and build capacity in recruiting Asian American immigrants with limited English proficiency. Sharing successes and lessons learned at multiple sites will contribute to developing more effective recruitment strategies for future aging research involving technology.

